# Impact of Vitamin D_3_ Deficiency on Phosphatidylcholine-/Ethanolamine, Plasmalogen-, Lyso-Phosphatidylcholine-/Ethanolamine, Carnitine- and Triacyl Glyceride-Homeostasis in Neuroblastoma Cells and Murine Brain

**DOI:** 10.3390/biom11111699

**Published:** 2021-11-15

**Authors:** Anna Andrea Lauer, Lea Victoria Griebsch, Sabrina Melanie Pilz, Daniel Janitschke, Elena Leoni Theiss, Jörg Reichrath, Christian Herr, Christoph Beisswenger, Robert Bals, Teresa Giovanna Valencak, Dorothea Portius, Heike Sabine Grimm, Tobias Hartmann, Marcus Otto Walter Grimm

**Affiliations:** 1 Experimental Neurology, Saarland University, 66421 Homburg, Germany; Anna.Lauer@uks.eu (A.A.L.); lea@ifuws.de (L.V.G.); pilz.sabrina1995@gmail.com (S.M.P.); daniel.janitschke@uks.eu (D.J.); elena.theiss@web.de (E.L.T.); heike.grimm@gmx.de (H.S.G.); 2 Department of Dermatology, Saarland University Hospital, 66421 Homburg, Germany; joerg.reichrath@uks.eu; 3 Department of Internal Medicine V-Pulmonology, Allergology, Respiratory Intensive Care Medicine, Saarland University Hospital, 66421 Homburg, Germany; christian.herr@uks.eu (C.H.); christoph.beisswenger@uks.eu (C.B.); robert.bals@uks.eu (R.B.); 4 Department of Biosciences, Paris Lodron University Salzburg, Hellbrunnerstrasse 34, 5020 Salzburg, Germany; Teresa.Valencak@vetmeduni.ac.at; 5 College of Animal Sciences, Zijingang Campus, Zhejiang University, Hangzhou 310058, China; 6 Nutrition Therapy and Counseling, Campus Gera, SRH University of Applied Health Science, 07548 Gera, Germany; Dorothea.Portius@srh.de; 7 Deutsches Institut für Demenzprävention, Saarland University, 66421 Homburg, Germany; tobias.hartmann@uks.eu; 8 Nutrition Therapy and Counseling, Campus Rheinland, SRH University of Applied Health Science, 51377 Leverkusen, Germany

**Keywords:** vitamin D hypovitaminosis, shotgun lipidomics, calcitriol, neurodegenerative diseases, phosphatidylcholine, phosphatidylcholine plasmalogen, lyso-phosphatidylcholine, carnitine, triacyl glyceride

## Abstract

Vitamin D_3_ hypovitaminosis is associated with several neurological diseases such as Alzheimer’s disease, Parkinson’s disease or multiple sclerosis but also with other diseases such as cancer, diabetes or diseases linked to inflammatory processes. Importantly, in all of these diseases lipids have at least a disease modifying effect. Besides its well-known property to modulate gene-expression via the VDR-receptor, less is known if vitamin D hypovitaminosis influences lipid homeostasis and if these potential changes contribute to the pathology of the diseases themselves. Therefore, we analyzed mouse brain with a mild vitamin D hypovitaminosis via a targeted shotgun lipidomic approach, including phosphatidylcholine, plasmalogens, lyso-phosphatidylcholine, (acyl-/acetyl-) carnitines and triglycerides. Alterations were compared with neuroblastoma cells cultivated in the presence and with decreased levels of vitamin D. Both in cell culture and in vivo, decreased vitamin D level resulted in changed lipid levels. While triglycerides were decreased, carnitines were increased under vitamin D hypovitaminosis suggesting an impact of vitamin D on energy metabolism. Additionally, lyso-phosphatidylcholines in particular saturated phosphatidylcholine (e.g., PC aa 48:0) and plasmalogen species (e.g., PC ae 42:0) tended to be increased. Our results suggest that vitamin D hypovitaminosis not only may affect gene expression but also may directly influence cellular lipid homeostasis and affect lipid turnover in disease states that are known for vitamin D hypovitaminosis.

## 1. Introduction

About 85% of the elderly population has vitamin D hypovitaminosis with vitamin D_3_ serum levels <50 nmol/L [1,2]. The insufficient levels of this fat-soluble vitamin could be attributed to their homebound lifestyle since vitamin D_3_ is synthesized in the human skin exposed to solar ultraviolet (UV) B radiation (290–315 nm) starting from 7-dehydrocholesterol [3]. This secosteroid, also called cholecalciferol, is hydroxylated in the liver by cytochrome P450 2R1 (CYP2R1) to 25-hydroxyvitamin D_3_ (Calcidiol, 25(OH) Vitamin D_3_). Calcidiol is further hydroxylated in the kidney by cytochrome P450 27B1 (CYP27B1), resulting in the formation of 1,25-dihydroxyvitamin D_3_ (Calcitriol, 25(OH)_2_ Vitamin D_3_), the active hormone, which exerts its biological effects via binding to the vitamin D receptor (VDR). Mediated by this nuclear hormone receptor superfamily member, forming a heterodimer with the retinoid X receptor, vitamin D_3_ regulates the transcription of genes containing a VDR response element [4].

The best-known biological function of vitamin D_3_ was found in the modulation of calcium and phosphorus homeostasis, being essential for bone mineralization. In this context, vitamin D_3_ deficiency is the cause for osteomalacia and osteoporotic pathophysiology in the elderly [5]. Numerous other functions of vitamin D_3_, for example immunomodulatory functions, were reported. Moreover, vitamin D_3_ could be associated with several other health disorders, such as cardiovascular disease, metabolic syndrome, insulin resistance, diabetes, different types of cancer, nonalcoholic fatty liver disease (NAFLD) as well as neurodegenerative and neuropsychiatric disease [6,7,8,9,10].

It was shown previously that vitamin D is associated with membrane fractions in rat kidney and based on these findings, a link of vitamin D_3_ actions with the function of cellular membranes was assumed [11]. Additionally, and concerning the role of vitamin D_3_ in calcium homeostasis, the calcium absorption by the small intestine is mediated by transporting calcium across biological membranes. In line with this, there is early evidence from the literature that 1,25-dihydroxyvitamin D_3_ influences phospholipid composition in rat kidney samples [12] or chick intestine [13]. Recently, the relevance of the VDR for liver lipid metabolism was shown in human hepatocytes using transcriptomic and metabolomic analyses [14]. Additionally, in the intestine, VDR-mediated changes in lipid homeostasis were observed in mouse studies [15,16]. Moreover, several observational and metabolic studies on vitamin D and lipid profiles from serum samples as well as randomized controlled trials were published, yet with inconsistent outcomes as reviewed in [17]. A recent clinical study examining the effects of vitamin D status on serum lipids in 1475 Chinese adults suggested a gender-dependent association between vitamin D deficiency and an elevated risk for dyslipidemia [18].

Based on the findings that enzymes involved in the synthesis of vitamin D_3_ and the VDR itself are expressed in brain tissue, the link between vitamin D_3_ and brain functions is obvious. The question whether vitamin D_3_ deficiency is associated with neurodegenerative disorders was the aim of our current research. In this context, recent studies could elucidate the molecular mechanisms of vitamin D and their role in Alzheimer’s disease [8,19]. Regarding 1,25-dihydroxyvitamin D_3_, an early study reported increased neuronal density after permanent treatment of rats with calcitriol for up to 12 months and suggested a function of vitamin D in the modulation of markers of brain aging [20]. An overview of the multiple brain functions and the neuro-psycho-pathophysiological role of calcitriol resulting from animal and cell culture studies is given in [21].

Most of the published studies investigated the effects of vitamin D_3_ on lipids in tissues such as kidney or intestine and in the context of neurological or chronic diseases, but little is known about the influence of this secosteroid on brain lipid metabolism. Moreover, the question if a direct effect of vitamin D_3_ on lipids may trigger the pathophysiology of several diseases that are known to be related to impairments in vitamin D_3_ status arises since numerous lipids are involved in the before mentioned diseases, as shown in Supplementary Figure S1 [22,23,24,25,26,27,28,29,30,31,32,33,34]. In the present study, we aimed at clarifying the role of vitamin D_3_ and VDR on lipid homeostasis in brain. Therefore, we use a targeted lipidomics profiling approach analyzing the most abundant lipid species in the human neuroblastoma cell line SH-SY5Y [35], a frequently used cellular model for studying neurodegenerative disorders such as Alzheimer’s disease [36,37,38]. Besides the fact that they are the main lipid species in brain and the used neuroblastoma cell line, we focused on lipids, which are known to be altered by AD, or lipids, which are indicated to modulate the disease progression. Moreover, lipids, which have a sufficient signal-to-noise ratio, can be unambiguously identified by shotgun lipidomics approaches utilizing multiple reaction monitoring. These lipids, 255 species in total, include phospholipids (phosphatidylcholines (PCaa), phosphatidylcholines-plasmalogens (PCae), lyso-phosphatidylcholines (lyso-PC)), and lipid species associated to the cellular energy metabolism and β-oxidation (carnitines and neutral lipids (triacyl glycerides (TAG)). Phosphatidylcholine (PC, 1,2-diacyl-sn-glycero-3-phosphocholine), also known under its trivial name lecithin, is the most abundant phospholipid in animals and an essential component of membrane bilayers. PC can exist in the diacyl form (PCaa) or in the alkyl-acyl form (PCae), also known as PC-plasmalogen. We compared SH-SY5Y cells treated with 1,25-dihydroxy vitamin D_3_ with cells incubated with the solvent control to simulate a condition of hypovitaminosis. To evaluate our findings of cell culture experiments in vivo, we performed this targeted shotgun lipidomics approach with brain samples of mice exposed to a 23% reduced level of 25-hydroxy vitamin D_3_.

## 2. Materials and Methods

### 2.1. Chemicals, Reagents and Standards

High performance liquid chromatography (HPLC-) grade pyridine, 1,25-dihydroxyvitamin D_3_, ammonium acetate and phenyl isothiocyanate (PITC) were acquired from Merck (Darmstadt, Germany). All other used chemicals in this study were purchased from Fisher Scientific (Schwerte, Germany), if not stated otherwise. Standards used for normalization in lipid analysis were acquired from Avanti Polar Lipids (06:0 PC (DHPC), 19:0 Lyso-PC, C18(Plasm)-18:1(d9) PC and 15:0-18:1(d7)-15:0 TG both contained in the Splash II Lipidomix Mass Spec Internal Standard, 08:0 PE) or from Supelco Analytical (octanoyl-l-carnitine d3, palmitoyl-l-carnitine d3).

### 2.2. Cell Culture and Calcitriol-Treatment

Human neuroblastoma SH-SY5Y wildtype cells were cultivated in Dulbecco´s Modified Eagle´s Medium (DMEM) containing 10% FBS (fetal bovine serum; GE Healthcare Life Sciences, Chalfont St. Giles, UK) and 0.1 mM nonessential amino acids in a humified incubator at 37 °C at 5% CO_2_. At a 90% confluence, the FBS content in the medium was reduced to 1% for 16 h to minimize the influence of vitamin D_3_ from serum. Afterwards, cells were incubated with 100 nM 1,25-dihydroxyvitamin D_3_ for 48 h while refreshing the incubation medium after 24 h. Solvent-control-treated cells were incubated with ethanol at a final concentration of 1‰, corresponding to the concentration in the incubation media, since 1,25-dihydroxyvitamin D_3_ was dissolved in ethanol.

### 2.3. Animal Experiments

Female C57BL/6 wt mice (Charles River, Sulzfeld, Germany) were six weeks old when the feeding experiment started. The mice were kept in a controlled environment with 50–60% humidity, a temperature between 20–22 °C and exposure to light from 7 a.m. until 7 p.m. Throughout the study, food and water were freely available. Mice were fed with control diet (C1000) or vitamin D deficient diet (C1017; Altromin, Lange, Germany) for 6–9 months. The control and vitamin D deficient diet were isocaloric and had identical contents of protein, carbohydrate, fiber and minerals. Compared to control-fed mice, the vitamin D deficient diet mice had a 23% reduced 25-hydroxyvitamin D_3_ serum level [39]. This vitamin D deficiency is in line with the situation in the elderly population [40,41] and under these experimental conditions we could show in previous studies that brain metabolism is affected, for example Aβ degradation due to neprilysin was decreased [19]. All animal experiments were approved by the “Landesamt für Soziales, Gesundheit und Verbraucherschutz of the State of Saarland” (reference number 17/2011) following the national guidelines for animal experimentation. Brains were dissected, washed in 0.9% sodium chloride, frozen in liquid nitrogen immediately and stored in liquid nitrogen until further analyses.

### 2.4. Sample Preparation

Vitamin D_3_- or control-incubated SH-SY5Y cells were harvested after 48 h at 4 °C. The conditioned medium was removed and used for analysis of cell viability via the Cytotoxicity Detection Kit (LDH) from Roche (Basel, Switzerland, Schweiz) according to manufacturer´s protocol. This colorimetric assay measured the lactate dehydrogenase (LDH) release from cells to quantify cell death and lysis after vitamin D incubations. Confluent cells were washed twice with ice-cold HPLC-grade water and harvested in 180 µL HPLC-grade water, followed by homogenization via Minilys (PEQLAB, Erlangen, Germany) using ceramic beads for 30 s on maximum intensity.

Mouse brain samples were slowly defrosted on ice and homogenized mechanistically in HPLC-grade water using Minilys (for detailed conditions, see above). We analyzed total brain homogenates in this study to potentially correlate effects on neurodegeneration with lipid changes. This is possible since we examined the same homogenates of the same mice, which were analyzed in a previous study, where we found APP processing to be affected by this mild to moderate vitamin D_3_ deficiency [19].

Sample protein concentration was determined using the bicinchoninic acid (BCA) assay according to Smith et al. [42]. Subsequently, homogenates were adjusted to the same protein amount (5 µg/µL for cell samples and 10 µg/µL for brain samples) by using HPLC-grade water.

### 2.5. Targeted Shotgun Mass Spectrometry

Prior to mass spectrometry analysis, lipids from a 100 µg sample were extracted using the solid/liquid lipid extraction method as described earlier in detail [43,44,45]. Afterward, shotgun lipidomics was performed on a 4000-quadrupole linear-ion trap (QTrap,) equipped with a Turbo Spray ion source from AB Sciex (Darmstadt, Germany) and couplet to an autosampler of the Agilent HPLC 1200 series (Santa Clara, CA, USA). Quantification of 174 different species of PCaa, PCae, lyso-PC, carnitines and TAG as well as 81 different species of PEaa, PEae and lyso-PE was carried out in technical triplicates using the Analyst 1.4.2 software from AB Sciex. The exact parameters of the lipid analysis performed in positive mode were defined in [44]. By calculating the ratio between the used lipid standards in presence of lipid extracts from control- and vitamin D_3_-treated cells or control- and vitamin D deficient mice, the potential matrix effects were calculated. Matrix effects are according to the IUPAC definition “the combine effect(s) of all components of the sample other than the analyte on the measurement of the quantity”, whereby suppression or enhancement were distinguished [46]. This means that the response for an analyte in mass spectrometry is not the same in a standard solution compared with the response of the same analyte in a biological matrix [47]. The change in this ratio was on average 1.87% for SH-SY5Y cellular samples and 1.80% for murine samples (see Supplementary Figure S2D,E).

### 2.6. Gene Expression Analysis

Total cellular RNA was extracted using TRIzol reagent according to manufacturer´s protocol and subsequently two micrograms of RNA were reverse-transcribed to generate complementary DNA (cDNA) via the High-Capacity cDNA Reverse Transcription Kit, as described by the manufacturer. Primers for RT-PCR, which was performed using Fast SYBR green Master Mix (Applied Biosystems, Forster City, CA, USA) on a PikoReal Real-Time PCR System (Thermo Fisher Scientific, Waltham, MA, USA), were IL-34 fwd 5′-AAT-CCG-TGT-TGT-CCC-TCT-TG-3′ and IL-34 rev 5′-CAG-CAG-GAG-CAG-TAC-AGC-AG-3′ as gene of interest and RN18S1 fwd 5′-GGA-GTA-TGG-TTG-CAA-AGC-TGA-3′ and RN18S1 rev 5′-ATC-TGT-CAA-TCC-TGT-CCG-TGT-3′ as housekeeping gene for normalization. Analysis of the data was carried out with the 2^−ΔΔCq^ method.

### 2.7. Analysis of Oxygen Consumption

The oxygen consumption of SH-SY5Y cells treated with 1,25-dihydroxyvitamin D_3_ as described above was measured with the Extracellular Oxygen Consumption Assay from Abcam (Cambridge, UK) according to manufacturer´s protocol. The multifunctional monochromator-based microplate reader Infinite M1000Pro from Tecan was used with excitation at 380 ± 20 nm and emission at 650 ± 20 nm (Tecan Group AG, Männedorf, Switzerland).

### 2.8. Data and Statistical Analysis

The Analyst 1.4.2. software from AB Sciex was used to extract counts per second for each MRM pair before each lipid was normalized to its respective lipid class internal standard. Afterwards, the means per triplicate for each lipid/standard ratio were formed per sample (at least five independent samples per control and treatment group). R (R Core Team 2020; Vienna, Austria; https://www.R-project.org/; accessed on 1 June 2021) was used to perform the statistical analysis and calculation of the p value for each lipid species, that are shown in the volcano plots, was carried out using the two-tailed Student´s t-test. Creation of the volcano plots was performed using the R package “EnhancedVolcano” (Kevin Blighe, Sharmila Rana and Myles Lewis (2020). version 1.6.0. https://github.com/kevinblighe/EnhancedVolcano; accessed on 1 June 2021). Fisher´s exact test was used to calculate if the increased/decreased lipid distribution significantly differed between the cellular incubations and mouse brain samples, respectively. Figures were created using CorelDRAW 11 (Corel Cooperation, Ottawa, ON, Canada). Error bars in the bar charts represent the standard error of the mean. Significance was set at * *p*  ≤  0.05, ** *p*  ≤  0.01 and *** *p*  ≤  0.001.

## 3. Results

To investigate if a deficit of calcitriol (1,25-dihydroxyvitamin D_3_), the active form of the fat-soluble vitamin D_3_, has an influence on lipid homeostasis in brain, we treated human neuroblastoma SH-SY5Y cells for 48 h with 100 nM 1,25(OH)_2_ vitamin D_3_ solved in ethanol and compared the changes in the lipid profile to that of cells treated with the solvent control under the same conditions. Results showed that calcitriol treatment had no effect on the viability of the neuroblastoma cells, as confirmed by performing a cytotoxicity detection assay (see Supplementary Figure S2A). A sufficient uptake of 1,25-dihydroxyvitamin D_3_ was verified by an increased gene expression of *IL-34*, whose transcription is known to be upregulated in SH-SY5Y cells treated with this active form of vitamin D_3_ [48] (see Supplementary Figure S2B). Moreover, the same targeted shotgun lipidomics approach was used to analyze the lipid profile of brain samples from mice with a 23% vitamin D deficiency due to a specialized diet compared to control-fed mice to evaluate if the effects observed in the cell culture can also be found in vivo. After extracting all lipids from the cellular and murine samples, 255 lipid species were semiquantitatively measured by shotgun mass spectrometry, including phospholipids (PCaa, PCae, Lyso-PC) and lipids involved in cellular energy metabolism (carnitines and neutral lipids (TAG)). Notably, using this method, the sum of the fatty acids bound to the stereospecific numbering 1 (sn-1) and sn-2 position of the glycerol backbone was detected. Yet, we cannot unambiguously distinguish between single fatty acids, which might be a caveat of our lipidomics approach. Normalization of the obtained data was done using internal standards of each lipid class of interest. Data were further analyzed as x-fold change and as changes in mol% compared to cells treated with 1,25(OH)_2_ vitamin D_3_ or, in case of the in vivo samples, compared to samples fed with the control diet. The observed relative changes in the lipid species are shown as volcano plots, in which the fold change (abscissa) is plotted against the *p*-value (ordinate). Two vertical lines mark the average standard error of the mean (SEM), and one horizontal line represents a *p*-value of 0.05, which was set as statistical significance. The corresponding bar chart for each lipid species is placed right to the volcano plot. Moreover, processed data dealing with saturation and chain length of the fatty acids bound to the backbone of the lipid are shown below the volcano plots and the bar chart. Since the definition of significance depends on the used statistical method (for example, which type one error correction) as well as on the sample number and since it has an arbitrary aspect, we included all lipid species in our study having a fold change greater than the average SEM of the corresponding lipid class but not reaching statistical significance. Moreover, it should be considered that the distribution of lipids within lipid classes sharing chemical similarities is relevant for both cellular and in vivo effects, beyond the significance of a single lipid species. Based on these intentions, we preferred volcano plots representing our results, allowing us to evaluate the distribution of lipid species within a lipid class without losing this information by just focusing on the significant parameters. In this context, we would like to point out that it has to be taken into consideration that these results should be verified in further studies.

### 3.1. Phosphatidylcholine (PCaa) Species

**Figure 1:**
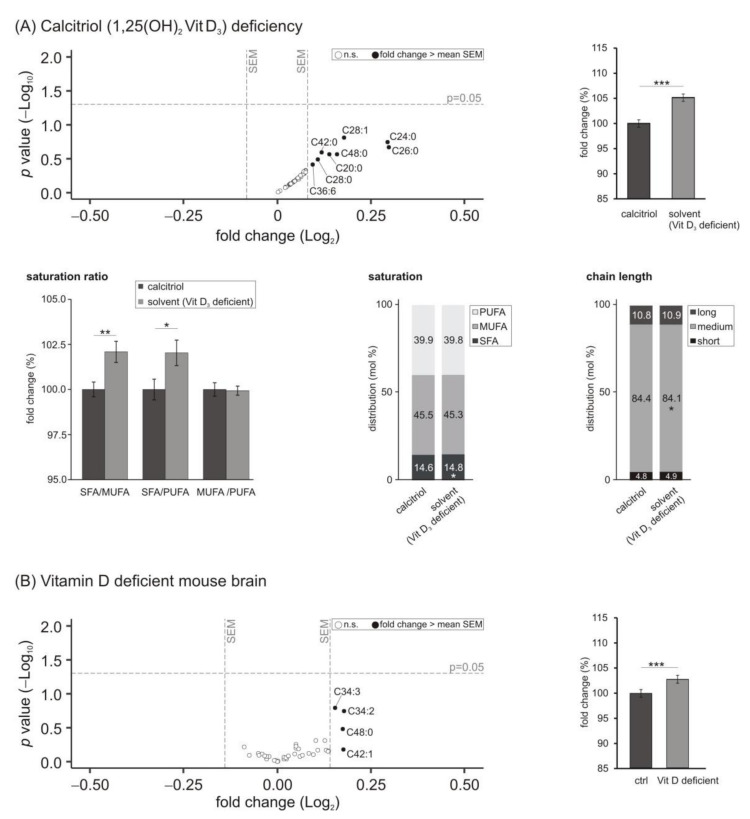
Effect of vitamin D_3_ deficiency on phosphatidylcholine (PCaa) species. (**A**) The levels of PCaa in SH-SY5Y wt cells incubated with the solvent control (ethanol) were compared to cells treated with 1,25-dihydroxy vitamin D_3_. In the volcano plot, the fold change (*x*-axis) of each of the 43 analyzed PCaa species (dots) was plotted against the corresponding *p*-value (*y*-axis). The two vertical lines represent the mean SEM. The horizontal line marks the *p* value of 0.05, which was set as statistical significance. Filled black dots symbolize PCaa species with a fold change greater than the mean SEM without reaching significance and empty dots represent species with a fold change within the mean SEM. The bar chart on the right shows the relative fold change of all measured PCaa species comparing calcitriol-treated with solvent-control-treated SH-SY5Y cells. Below the volcano plot, ratios indicating the saturation state of the fatty acids within the PCaa species and the distribution of saturation and chain length in mol% are shown. (**B**) Analyzed PCaa species in brain samples of vitamin D deficient mice compared to control-fed mice in a volcano plot and relative fold changes of all measured PCaa species in a bar chart. Error bars represent the standard error of the mean (SEM). Statistical significance was set as * *p* ≤ 0.05, ** *p* ≤ 0.01 and *** *p* ≤ 0.001; n.s. not significant.

The major phospholipid components ubiquitously found in cell membranes are glycerophospholipids. Moreover, phosphatidylcholine (PC; 1,2-diacyl-*sn*-glycero-3-phosphocholine) represents about 9–10% of dry matter of the human brain and 30–35% of total phospholipid [49]. In our study, a deficit of calcitriol in SH-SY5Y cells led to increased levels of PCaa species (Figure 1A). This becomes obvious since all measured PCaa species were located on the right side of the volcano plot. Some of them were increased with a fold change greater than the average SEM, but none of them reached statistical significance. Nevertheless, it is interesting that the whole lipid class was increased in cells deficient in the active form of vitamin D_3_. On average, the PCaa species were elevated to 105.1 ± 0.7% (*p* = 0.000), as shown in the bar chart (Figure 1A). Detailed analyses of the data revealed differences in the ratios of PCaa species containing saturated fatty acids (SFA), monounsaturated fatty acids (MUFA) and polyunsaturated fatty acids (PUFA). The SFA/MUFA and SFA/PUFA ratios were significantly increased (SFA/MUFA: 102.1 ± 0.6%, *p* = 0.006; SFA/PUFA: 102.0 ± 0.7%, *p* = 0.033), due to increased levels of PCaa species containing a SFA (14.6 to 14.8 mol%, *p*= 0.012) and a slight decrease in MUFA- and PUFA-containing species (MUFA: 45.5 to 45.3 mol%, *p* = 0.0954; PUFA: 39.9 to 39.8 mol%, *p* = 0.0382). In line with this, reduced levels of medium chain fatty acid containing PCaa species (C32:X–C36:X) in SH-SY5Y cells deficient in calcitriol were observed.

Interestingly, a similar trend of increased PCaa species due to calcitriol deficiency could be observed in the vitamin D deficient murine brain samples (see Figure 1B). Most measured PCaa species tended to be elevated and an increase to 102.9 ± 8.3% (*p* = 0.000) could be detected in the means, which was less significant than the results obtained from the cell culture experiments. Moreover, also in the in vivo samples, PCaa species containing medium chain fatty acids were slightly decreased from 76.6 to 76.3 mol% (data not shown).

### 3.2. Phosphatidylcholine Plasmalogens (PCae) Species

**Figure 2:**
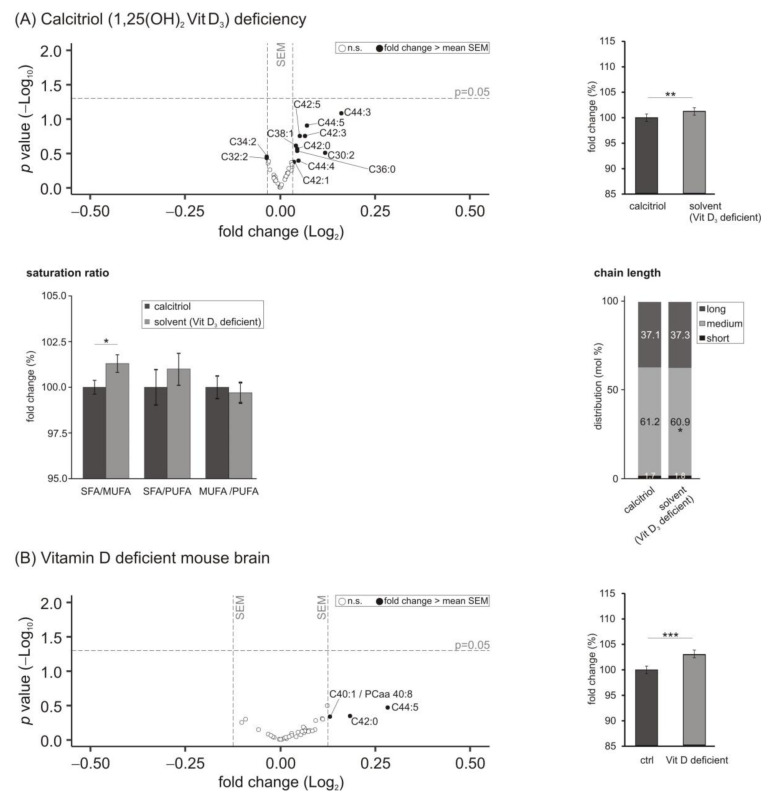
Effect of vitamin D_3_ deficiency on phosphatidylcholine plasmalogens (PCae) species. (**A**) The levels of PCae in SH-SY5Y wt cells incubated with the solvent control (ethanol) were compared to cells treated with 1,25-dihydroxy vitamin D_3_. In the volcano plot, the fold change (*x*-axis) of each of the 39 analyzed PCae species (dots) was plotted against the corresponding *p*-value (*y*-axis). The volcano plots are structured as described in detail in the legend of Figure 1. The bar chart on the right shows the relative fold change of all measured PCae species comparing calcitriol-treated with solvent-control-treated SH-SY5Y cells. Below the volcano plot, ratios dealing with the saturation state of the fatty acids within the PCae species and the distribution of chain length in mol% are shown. (**B**) Analyzed PCae species in brain samples of vitamin D deficient mice compared to control-fed mice in a volcano plot and relative fold changes of all measured PCae species in a bar chart. Error bars represent the standard error of the mean (SEM). Statistical significance was set as * *p* ≤ 0.05, ** *p* ≤ 0.01 and *** *p* ≤ 0.001; n.s. not significant.

Plasmalogens (PCae; 1-alkyl, 2-acyl-*sn*-glycero-3-phosphorylcholine), constituting up to 22% of the total phospholipids in human brain, are membrane glycerophospholipids containing a fatty alcohol with a vinyl-ether bond at the sn-1 position [50]. We found that in human neuroblastoma cells deficient in calcitriol the measured PCae species tended to increase, since these lipids with changes greater than the average SEM were upregulated. In total, a slight but significant elevation in PCae species to 101.3 ± 0.5% (*p* = 0.008) was detected when comparing cells treated with 1,25-dihydroxy vitamin D_3_ with cells incubated with the solvent control (Figure 2A). Comparable to the finding in PCaa species, the SFA/MUFA and the SFA/PUFA ratios were elevated, with the SFA/MUFA ratio reaching statistical significance (SFA/MUFA: 101.3 ± 0.5%, *p* = 0.0433; SFA/PUFA: 101.0 ± 0.9%, *p* = 0.454). These changes observed in the saturation ratios could be explained by increased SFA-containing PCae species and decreased MUFA-containing PCae species: SFA from 12.5 to 12.6 mol% with *p* = 0.286 and MUFA from 31.9 to 31.7 mol% with *p* = 0.0462 (data not shown). Regarding the chain lengths of the fatty acids composing the measured PCae species, we observed that PCae species containing medium chain fatty acids (C32:X–C36:X) were significantly decreased from 61.2 to 60.9 mol% (*p* = 0.0194).

In line with the findings of the cell culture-based experiments, the levels of PCae species were increased to 103.1 ± 0.8% (*p* = 0.001) in brain samples of mice with mild vitamin D hypovitaminosis (Figure 2B). Moreover, ratios of SFA/MUFA and SFA/PUFA tended to be increased (SFA/MUFA to 104.7 ± 2.8%, *p* = 0.282 and SFA/PUFA to 104.6 ± 2.6%, *p* = 0.247), similar to the significant results in SH-SY5Y cells. SFA-containing PCae species showed an elevated trend due to the vitamin D deficit from 32.8 to 33.8 mol% (*p* = 0.257), while MUFA and PUFA fatty-acid-containing plasmalogen species tended to be reduced (MUFA from 34.2 to 33.7 mol%, *p* = 0.387 and PUFA from 33.0 to 32.5 mol%, *p* = 0.329). The findings relating to the saturation of PCae species failed to reach statistical significance. This might be explained by the lower sample size of the in vivo material compared to the cell culture treatments. Comparable to the effects of hypovitaminosis D in neuroblastoma cells, a similar trend of reduced medium-chain fatty acids (C32:X–C36:X) in PCae species could also be observed in the in vivo samples (from 44.0 to 42.9 mol%, *p* = 0.320).

### 3.3. Lyso-Phosphatidylcholine (Lyso-PC) Species

**Figure 3:**
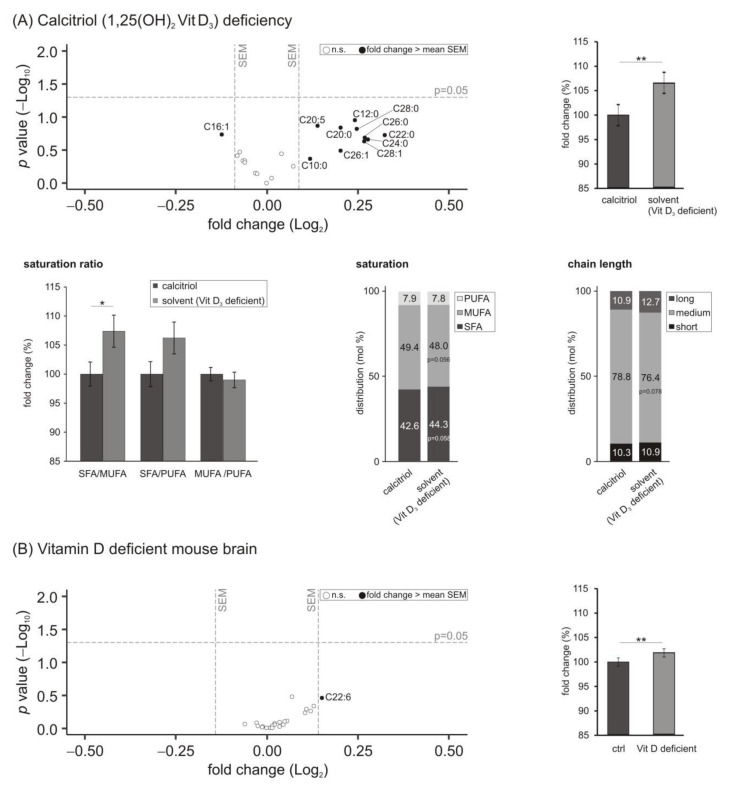
Effect of vitamin D_3_ deficiency on lyso phosphatidylcholine (lyso-PC) species. (**A**) The levels of lyso-PC in SH-SY5Y wt cells incubated with the solvent control (ethanol) were compared to cells treated with 1,25-dihydroxy vitamin D_3_. In the volcano plot the fold change (*x*-axis) of each of the 22 analyzed lyso-PC species (dots) was plotted against the corresponding *p*-value (*y*-axis). The volcano plots are structured as described in detail in the legend of Figure 1. The bar chart on the right shows the relative fold change of all measured lyso-PC species comparing calcitriol-treated with solvent-control-treated SH-SY5Y cells. Below the volcano plot, ratios dealing with the saturation state of the fatty acids within the lyso-PC species and the distribution of chain length in mol% are shown. (**B**) Analyzed lyso-PC species in brain samples of vitamin D deficient mice compared to control-fed mice in a volcano plot and relative fold changes of all measured lyso-PC species in a bar chart. Error bars represent the standard error of the mean (SEM). Statistical significance was set as * *p* ≤ 0.05 and ** *p* ≤ 0.01; n.s. not significant.

Lyso-phosphatidylcholines (Lyso-PC; 1- or 2-acyl-*sn*-glycero-3-phosphorylcholine) are derived from phosphatidylcholines due to the catabolic activity of the phospholipase A_2_ (PLA_2_) and can be found in the nervous system [49]. In our study, nine out of the ten analyzed lyso-PC species that showed a fold change greater than the mean SEM were upregulated due to a deficiency of calcitriol (Figure 3A). On average, the levels of lyso-PC species were increased significantly to 106.7 ± 2.3% (*p* = 0.006) comparing solvent-control-treated cells to cells incubated with 1,25-dihydroxyvitamin D_3_. Similar to the observed effect of hypovitaminosis D on the saturation of PCaa species, the saturation state of lyso-PC fatty acids was influenced under these experimental conditions. The ratio of SFA/MUFA was increased significantly to 107.4 ± 2.8% (*p* = 0.0456) and the SFA/PUFA ratio tended to be elevated (106.2 ± 2.7%, *p* = 0.092), while the ratio of MUFA/PUFA remained unchanged. The observations could be explained by increased SFA and decreased MUFA levels (SFA: 42.6 to 44.3 mol%, *p* = 0.058; MUFA: 49.4 to 48.0 mol%, *p* = 0.056). Moreover, PUFA levels tended to decrease (7.9 to 7.8 mol%, *p* = 0.323) with X:2 fatty acids significantly reduced from 2.5 to 2.4 mol% (*p* = 0.020). In line with the findings of PCaa species, lyso-PC species containing medium-chain fatty acids (C16:X–C20:X) tended to be reduced in SH-SY5Y cells treated with the solvent control compared to those incubated with calcitriol (78.8 to 76.4 mol%, *p* = 0.078).

In the vitamin D deficient in vivo mouse brain samples, a similar but weaker effect of vitamin D hypovitaminosis on lyso-PC levels with an increase to 102.8 ± 0.9% (*p* = 0.003) could be observed (Figure 3B).

### 3.4. Phosphatidylethanolamine (PE) Species

**Figure 4:**
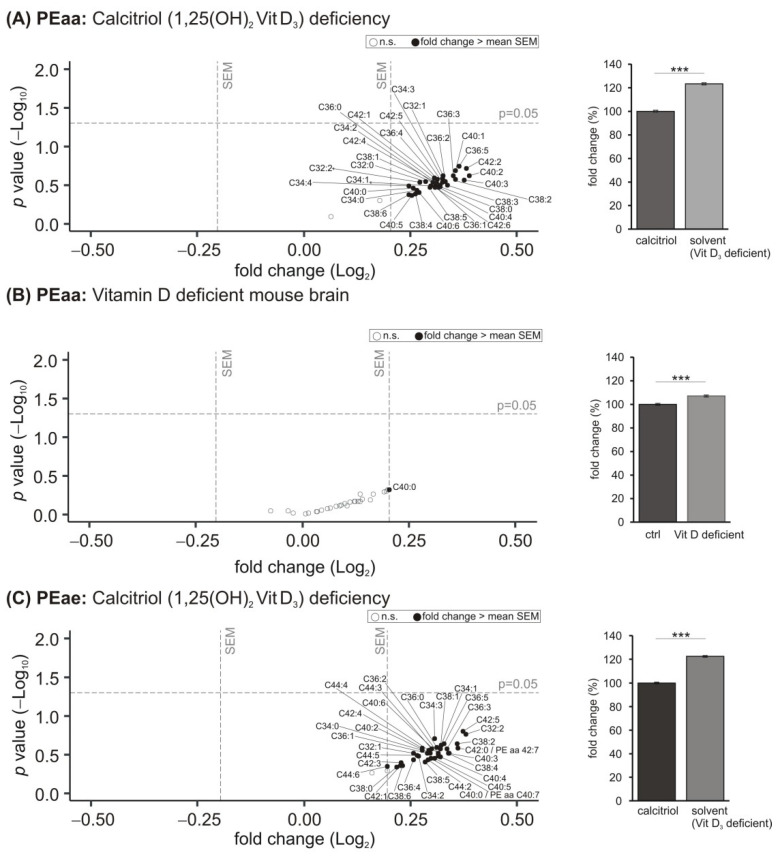

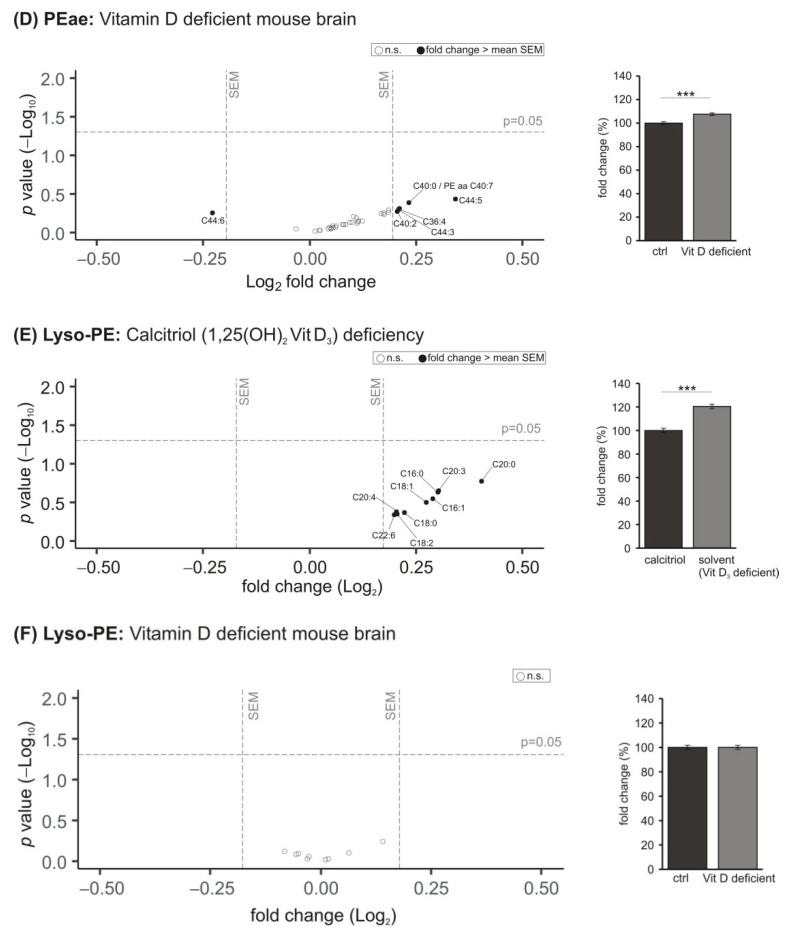
Effect of vitamin D_3_ deficiency on phosphatidylethanolamine (PE) species. (**A**) The levels of PEaa in SH-SY5Y wt cells incubated with the solvent control (ethanol) were compared to cells treated with 1,25-dihydroxy vitamin D_3_. In the volcano plot, the fold change (*x*-axis) of each of the 35 analyzed PEaa species (dots) was plotted against the corresponding *p*-value (*y*-axis). The volcano plots are structured as described in detail in the legend of Figure 1. The bar chart on the right shows the relative fold change of all measured PEaa species comparing calcitriol-treated with solvent-control-treated SH-SY5Y cells. (**B**) Analyzed PEaa species in brain samples of vitamin D deficient mice compared to control-fed mice in a volcano plot and relative fold changes of all measured PEaa species in a bar chart. Besides PEaa, also levels of PEae (**C**,**D**) and Lyso-PE species (**E**,**F**) under hypovitaminosis D3 conditions were examined. Error bars represent the standard error of the mean (SEM). Statistical significance was set as *** *p* ≤ 0.001; n.s. not significant.

Phosphatidylethanolamines (1,2-diacyl-sn-glycero-3-phosphoethanolamine, PE) are considered as the second most abundant phospholipids after PC. Moreover, they represent frequently one of the major lipid components of membranes. Similar to PC species, PE can exist in diacyl (PEaa) or alkyl-acyl (PEae/plasmalogens) forms. Under the vitamin D_3_ deficient conditions in our study, largely all PEaa species were found to be upregulated greater than the mean SEM in the analyzed human neuroblastoma cell line (Figure 4A). A significant mean increase to 123.3 ± 0.8% (*p* ≤ 0.001) was observed. A similar elevated trend could be found in the murine brain samples, but only one species (PEaa C40:0) showed an effect strength greater than the mean SEM, which could be explained by the smaller sample size of the in vivo material (Figure 4B). This increase to 107.1 ± 0.8% (*p* ≤ 0.001) was also significant. Regarding PE plasmalogens, we found similar results as observed for PEaa species before: 35 out of the 37 analyzed PEae species tended to be increased in vitamin D_3_ deficient SH-SY5Y cells, resulting in a significant mean elevation to 122.4 ± 0.7% (*p* ≤ 0.001) (Figure 4C). A similar trend was detected in the murine brain samples with the exception that two PEae species, including PEae C44:6, tended to be reduced (Figure 4D). A significant mean increase to 107.5 ± 1.1% (*p* ≤ 0.001) was observed. Moreover, the nine detected lyso-PE species were increased greater than the mean SEM in SH-SY5Y cells deficient in vitamin D_3_, resulting in a significant mean elevation to 120.4 ± 1.9% (*p* ≤ 0.001) (Figure 4E). This effect could not be detected in the murine brain samples (Figure 4F).

### 3.5. Lipid Species Involved in Cellular Energy Metabolism and β-Oxidation

**Figure 5:**
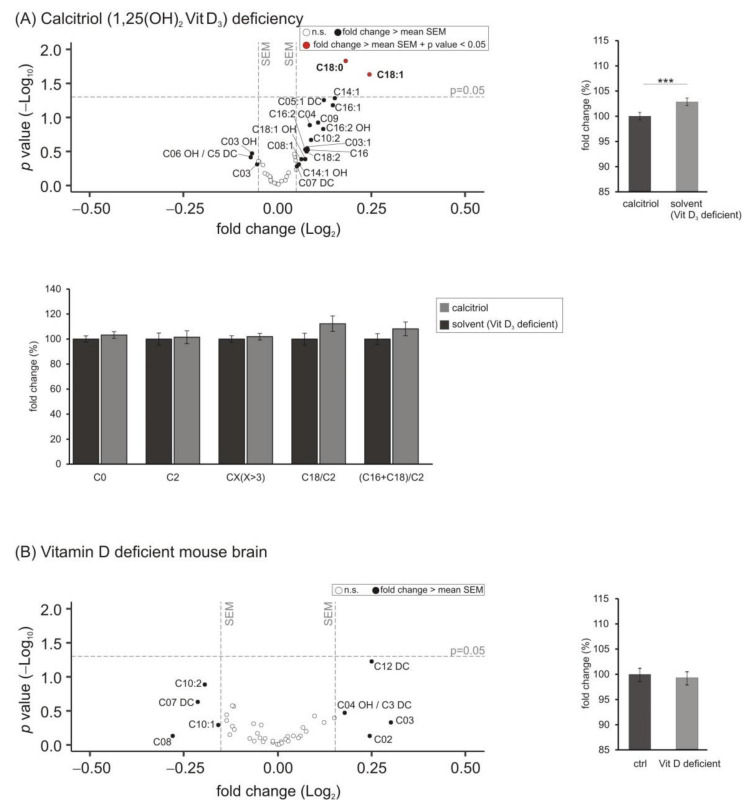
Effect of vitamin D_3_ deficiency on carnitine species. (**A**) The levels of carnitines in SH-SY5Y wt cells incubated with the solvent control (ethanol) were compared to cells treated with 1,25-dihydroxy vitamin D_3_. In the volcano plot, the fold change (*x*-axis) of each of the 41 analyzed carnitine species (dots) was plotted against the corresponding *p*-value (*y*-axis). The volcano plots are structured as described in detail in the legend of Figure 1. The bar chart on the right shows the relative fold change of all measured carnitine species comparing calcitriol-treated with solvent-control-treated SH-SY5Y cells. The bar chart below the volcano plot shows the changes in C0, C2, CX (with X > 3), C18/C2- and (C16 + C18)/C2 ratio in SH-SY5Y cells deficient in vitamin D_3_ compared to calcitriol-treated cells. (**B**) Analyzed carnitine species in brain samples of vitamin D deficient mice compared to control-fed mice in a volcano plot and relative fold changes of all measured carnitine species in a bar chart. Error bars represent the standard error of the mean (SEM). Statistical significance was set as *** *p* ≤ 0.001; n.s. not significant.

Besides phosphoglycerides, also carnitines, playing a major role in fatty acid transport from the cytosol to the mitochondria across the inner mitochondrial membrane for subsequent β-oxidation of fatty acids esterified to carnitines, were included in our study. We determined the levels of free l-carnitine (C0), acetyl-carnitine (C2) and acyl-carnitine (CX with X > 3). Figure 5 shows that a deficit of calcitriol resulted in significantly increased carnitine levels to 102.9 ± 0.8% (*p* = 0.001) with levels of carnitine C18:0 and C18:1 significantly upregulated (C18:0 to 113.4 ± 2.9%, *p* = 0.015 and C18:1 to 118.4 ± 5.2%, *p* = 0.023) (Figure 5A). Moreover, the carnitine species C03 and C03 OH were downregulated with a fold change greater than the mean SEM without reaching statistical significance (C03 to 96.3 ± 3.5%, *p* = 0.493 and C03OH to 95.4 ± 3.9%, *p* = 0.337). These findings of the cell culture experiments could not be observed in murine brain samples deficient in vitamin D, since no change in the 41 analyzed carnitine species could be detected (Figure 5B) (mean: 99.7 ± 1.4%, *p* = 0.816).

**Figure 6:**
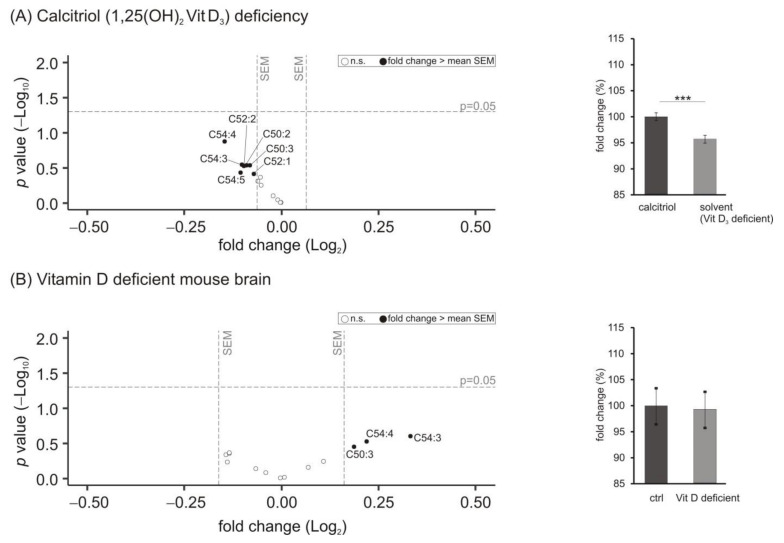
Effect of vitamin D_3_ deficiency on triacyl glycerides (TAG) species. (**A**) The levels of TAG in SH-SY5Y wt cells incubated with the solvent control (ethanol) were compared to cells treated with 1,25-dihydroxy vitamin D_3_. In the volcano plot, the fold change (*x*-axis) of each of the 14 analyzed TAG species (dots) was plotted against the corresponding *p*-value (*y*-axis). The volcano plots are structured as described in detail in the legend of Figure 1. The bar chart on the right shows the relative fold change of all measured TAG species comparing calcitriol-treated with solvent-control-treated SH-SY5Y cells. (**B**) Analyzed TAG species in brain samples of vitamin D deficient mice compared to control-fed mice in a volcano plot and relative fold changes of all measured TAG species in a bar chart. Error bars represent the standard error of the mean (SEM). Statistical significance was set as *** *p* ≤ 0.001; n.s. not significant.

Besides carnitines, also neutral glycerides, especially triacyl glycerols (TAG), are an important marker of cellular energy metabolism since they serve as storage by the formation of lipid droplets. Additionally, fatty acids derived from TAG are used for energy production in the mitochondria, where they were transferred to carnitines. In our study, comparing SH-SY5Y cells incubated with 1,25-dihydroxy vitamin D_3_ with cells deficient in calcitriol treated with the solvent control, all of the 14 analyzed TAG species tended to be decreased (Figure 6A). On average, the levels of TAG were significantly reduced to 95.7 ± 0.8% (*p* = 0.000).

Levels of these neutral lipids were not changed in brain samples of mice with a mild to moderate vitamin D hypovitaminosis. In contrast to the findings in the cell-based experiments, those TAG species with a fold change greater than the average SEM tended to be increased, as shown in the volcano plot (Figure 6B).

## 4. Discussion

Recent studies provide growing evidence for an important role of vitamin D_3_ in the regulation of the lipid homeostasis [11,12,13,14,15,16,17,18]. Most of the available studies analyzed this suggested link in the gastrointestinal system, kidney cells or tissue, but we lack information on such an association of vitamin D_3_ and lipid metabolism in the brain. Brain lipid homeostasis is an essential component for the emergence of neurodegenerative diseases, e.g., Alzheimer´s disease (AD): the cleavage of the amyloid-precursor-protein (APP), that results in the cerebral accumulation of the neurotoxic amyloid -β(Aβ) to senile Aβ plaques, one of the characteristic hallmarks of AD, takes place in the biological membranes, since APP is a type I transmembrane protein [51]. Furthermore, the proteases involved in this proteolytic processing, named α-, β- and γ-secretase, are membrane-tethered proteins with the γ-secretase cleaving APP within the hydrophobic membrane environment [52,53]. Thus, the question arises, if vitamin D_3_ is able to trigger the pathophysiology of AD by influencing the homeostasis of the lipids involved in the occurrence and the progression of this neurodegenerative disorder.

The occurrence of AD increases dramatically with the age as shown in a study from the United States reporting that 3% of people aged between 65 and 74 years have a diagnosis of AD dementia, while this number rises to 32% for people of 85 years or older [54]. Based on the fact that about 85% of the elderly population has insufficient vitamin D_3_ levels in blood serum, several existing studies suggest a potential link between vitamin D_3_ hypovitaminosis and AD. In this context, several negative effects of vitamin D_3_ deficiency with respect to neurodegenerative diseases and also several neuroprotective properties of a sufficient vitamin D_3_ status relating to the pathophysiological processes associated with AD were found [8,10,19,39]. Besides these recent findings, detailed information about a possible link between vitamin D_3_ and lipid species, which play key roles in the cellular membrane and that influence the proteolytic processing of APP, are still missing. One reason for that could be that the technique of lipidomics is not widely used in comparison to other “omics” technologies since labor-intensive workflows and diverse instruments are necessary to analyze the immense complexity of the lipidome, including thousands of different lipid species.

In our present study, we performed a detailed semiquantitative lipidomics approach investigating changes in lipid species known to be affected in AD pathophysiology in a neurocellular and a murine model for D_3_ hypovitaminosis. Regarding the murine samples, it must be mentioned as a limitation of our study that we examined total brain homogenates, which makes it impossible to distinguish between the different brain regions. We focused on changes in three lipid classes (phospholipids, carnitines and neutral lipids) since different lipid classes may mediate diverse biological effects. Moreover, we condensed our data to get insights into the molecular entities, for example the saturation or chain lengths of the fatty acids conjugated to the backbone. In general, increased levels of phospholipids and simultaneously changes in lipids involved in cellular energy metabolism were found, which will be discussed in detail in the following paragraphs.

As already mentioned in the results section, utilizing a shotgun lipidomics approach has, per se, some limitations, which were discussed in the review from Fong-Fu Hsu [55]. Briefly, we cannot distinguish between the fatty acids in the sn-1 and sn-2 positions or between (stereo-) isomers and lipids, which have the same fragmentation pattern in addition to the same Q1 mass (mother ion). Therefore, we focused on lipids which were, to our knowledge, not highly affected by other isobaric lipids or which showed a different fragmentation pattern in the Q3 scan. These lipids and the according MRMs were published previously, e.g., in [44,56,57,58].

Regarding phosphatidylcholine (PCaa) species, studies reported alterations in the PC metabolism in plasma samples of individuals suffering from AD [59]. Moreover, a recent study from Blasko et al., which will be discussed in detail later, described changed plasma phosphatidylcholines as possible prognostic biomarkers for the progression of AD. They observed five phospholipid species to be significantly increased in their conversion study dealing with patients with progression from cognitive health to AD [60]. In our present study, the PCaa levels were significantly increased in the cellular model of hypovitaminosis D_3_ and this finding could be verified in vivo since PCaa levels were also upregulated in brain samples of vitamin D deficient mice. Interestingly, in the neuroblastoma cell line every single measured species was upregulated, suggesting a direct influence of calcitriol on this lipid class. Moreover, the PCaa species found to be upregulated under hypovitaminosis D conditions share structural similarities with those found by Blasko and colleagues in plasma samples of 48 mild cognitive impairment (MCI) patients compared to healthy subjects in a 7–9 year follow up study: PCaa C32:1, PCaa C34:1 and PCaa C42:1 [60] in patients converting from MCI to AD diagnosis compared to PCaa C34:2, PCaa C34:3, PCaa C42:0 and PCaa C42:1 mediated through a vitamin D deficit (see Figure 1). However, it must be mentioned as a caveat in this context that there are at least two parameters in every lipid species, which can be compared: the chain length and the degree of saturation. Therefore, lipid species with different length and saturation should be considered different. Nevertheless, these results suggested a direct influence of vitamin D_3_ on lipid homeostasis involved in the progression of AD. In line with this evidence, a recent study found the VDR to be relevant for lipid metabolism in cultured human hepatocytes, especially for phospho- and glycerolipid homeostasis. The authors reported significant alterations in PCs due to VDR activation by calcitriol, such as, for example, elevated levels of PC(16:0/14:1) and simultaneously reduced levels of PC(20:4/20:3). Since some PC species appeared increased but others were decreased, the authors conclude that vitamin D_3_ may induce a remodeling of the phosphatidylcholine pool. Additionally, they performed a transcriptomic analysis revealing that one fifth of the VDR responsive genes were related to lipidic pathways such as glycerolipid- and phospholipid metabolism or the uptake of fatty acids. In line with this, they reported a coordinated gene response of these genes due to VDR activation by calcitriol [14]. Moreover, we found the expression of genes known to play a role in lipid metabolism altered under mild to moderate vitamin D deficiency in a previous study [8]. In the present study, the expression of IL-34, a gene, whose transcription is known to be upregulated by incubating SH-SY5Y cells with 100 nM 1,25(OH)2D3, is 1.5-fold increased under the experimental conditions (see Supplementary Figure S2B). This genomic action of vitamin D_3_ might be a molecular perspective of the changes observed in cellular lipids with vitamin D deficiency in our study and should be examined in further studies regarding genes involved in the metabolism of the lipid species found to be affected by a calcitriol deficiency. Additionally, nongenomic effects of 1,25(OH)_2_D_3_ at cellular membranes have been observed recently, representing a further molecular perspective of the changed cellular lipids, which should be the subject of further research [61].

Thorough analyses of our data revealed that SFA-containing PCaa species were significantly upregulated due to calcitriol deficiency. Correspondingly, MUFA- and PUFA-containing lipids tended to be reduced (see Figure 1). Similar findings were reported in a recent study analyzing fatty acid metabolism in a vitamin D_3_ deficient rat model. The plasma total SFA levels were higher in the hypovitaminosis group while levels of MUFA were lower [62]. Based on these findings, a possible influence of calcitriol on desaturases in the brain could be subject of further research, especially since the presence of SFAs may disrupt the structure of the cell membrane and may decrease its fluidity [63]. Membrane fluidity is negatively associated with the molecular mechanisms of AD since an elevated membrane fluidity, for example, due to enrichment of the cellular membrane with PUFAs, promotes the nonamyloidogenic cleavage of APP by the α-secretase [64]. These findings might suggest that vitamin D_3_ broadly affects APP homeostasis by influencing phospholipid metabolism. Moreover, phospholipids are some of the main components of lipid rafts (LR) from the cell membrane and the LR structural integrity and function depends, at least in part, on them. LR are dynamic structures and important for cellular processes such as lipid/protein sorting, signal transduction or cell adhesion, for example [65]. LR has been associated with neurodegenerative diseases such as AD, since amyloidogenesis, the production of the neurotoxic Aβ peptide, is related to the LR lipid composition [66]. The elevated levels of saturated fatty acids under the hypovitaminosis D_3_ conditions in our study could suggest an increased occurrence of LR in the membrane, since combinations of sphingolipids, cholesterol and saturated fatty acids are characteristic for LR. Interestingly, amyloidogenic processing caused by β- and γ-secretase is reported to be associated with LR [67]. As SFAs are major compounds in LR, one might speculate that the lipid alterations, which are due to vitamin D deficiency causes a shift from nonamyloidogenic processing to amyloidogenic processing. Additionally, LR were reported to be platforms for interactions of Aβ and ApoE or tau and to trigger the formation of Aβ fibrils [68,69].

In our study, levels of phosphatidylcholine-plasmalogens (PCae) were significantly increased in both cellular and murine hypovitaminosis D samples. In line with the alterations in the PCaa species, the fatty acid ratios SFA/MUFA and SFA/PUFA were changed and PCae species containing medium-chain fatty acids (C32:X–C36:X) were significantly decreased due to vitamin D_3_ deficiency. This increase in PCae could be caused by their anti-neuroinflammatory and anti-amyloidogenic properties [70]. As the negative effects of an insufficient vitamin D_3_ supply on inflammatory processes in the brain and Aβ metabolism have been reported [19,39,71], the observed increase in PCae species in cellular and murine hypovitaminosis samples could be interpreted as a possible defense mechanism.

Lyso-phosphatidylcholines species are mainly derived from the turnover of PC by the phospholipase A_2_ (PLA_2_) and represent the transport form of lipids. In the literature, lyso-PCs are described to be positively linked with neurodegenerative and cardiovascular diseases [72]. Moreover, this lipid class is linked to multiple sclerosis due to its proinflammatory properties [73]. Regarding AD, it was reported that lyso-PCs elevate the oligomer formation process of the Aβ_1-42_ peptide and thereby increase neuronal apoptotic death. Inhibition of lyso-PC synthesis thus has been suggested as a therapeutic target for the treatment of AD [74]. In this context, the observed increase in lyso-PC levels in our cellular and murine model of vitamin D_3_ hypovitaminosis suggests a link between a deficit of this secosteroid and related pathophysiology during AD.

As for lipids involved in cellular energy metabolism and β-oxidation, alterations due to calcitriol deficiency were detected in SH-SY5Y cells. Several lines of evidence indicate that in AD brain energy metabolism is impaired [75,76]. However, most publications refer to an altered glucose metabolism. It might be speculated that because of these and decreased energy supply by glucose metabolism, β-oxidation of fatty acids become of more importance in AD. Interestingly, it has been reported that mitochondrial oxygen consumption rate is increased upon 1,25-dihydroxy vitamin D_3_ treatment in human skeletal muscle cells, which is in line with other studies reporting that vitamin D deficiency decreases oxygen consumption and disrupts mitochondrial function [77,78]. Similar results were found under our experimental conditions in SH-SY5Y cells. Decreased oxygen consumption was found in cells with decreased vitamin D_3_ levels (see Supplementary Figure S2C). In this context, the significant increase of the acyl-carnitines C18 and C18:1 might be explained; C18 and C18:1 might accumulate due to a decreased turnover in β-oxidation and mitochondrial function. This is in line with the increase in the C18/C2 or (C16 + C18)/C2 ratio, further arguing for an impairment of fatty acid turnover in β-oxidation. In this context, it must be pointed out that due to its short half-life, the C18/C2- and (C16 + C18)/C2 ratio represents a snapshot rather than potential accumulative effects such as changes in the lipid homeostasis. However, we would like to point out that most of the effects were only observed in vitro in SH-SY5Y cells; in mouse deficient brain none of the effects reached a significant level and effect size was mainly in the standard deviation. Therefore, the experimental feeding conditions with very mild vitamin D deficiency seems not be sufficient to provoke effects observed in cell culture, and effects under these conditions with respect to mitochondrial function should not be overinterpreted.

The significant decrease in total TAG levels observed in SH-SY5Y cells deficient in calcitriol are well in line with the literature. A study performed in cultured human hepatocytes reported an intracellular accumulation of TAG when activating the VDR using calcitriol and thereby analyzing the opposite condition compared to our study [14]. Moreover, an animal study provided indications for an important role of the intestinal VDR in systemic lipid homeostasis. Vdr^−/−^ mice exhibited a decreased capacity to harvest TAG from the circulation and additionally these mice were protected from diet-induced obesity and hepatosteatosis, which is caused by excessive TAG accumulation in the liver [15]. These findings were in line with a study investigating the role of the VDR in nonalcoholic fatty liver disease (NAFLD). The authors reported a protection of high fat diet-fed apoE^−/−^ mice against fatty liver, dyslipidemia and insulin resistance due to deletion of the VDR. Concluding, they suggest that therapeutic inhibition of liver VDR may have beneficial effects regarding steatosis in early NAFLD [79].

**Figure 7:**
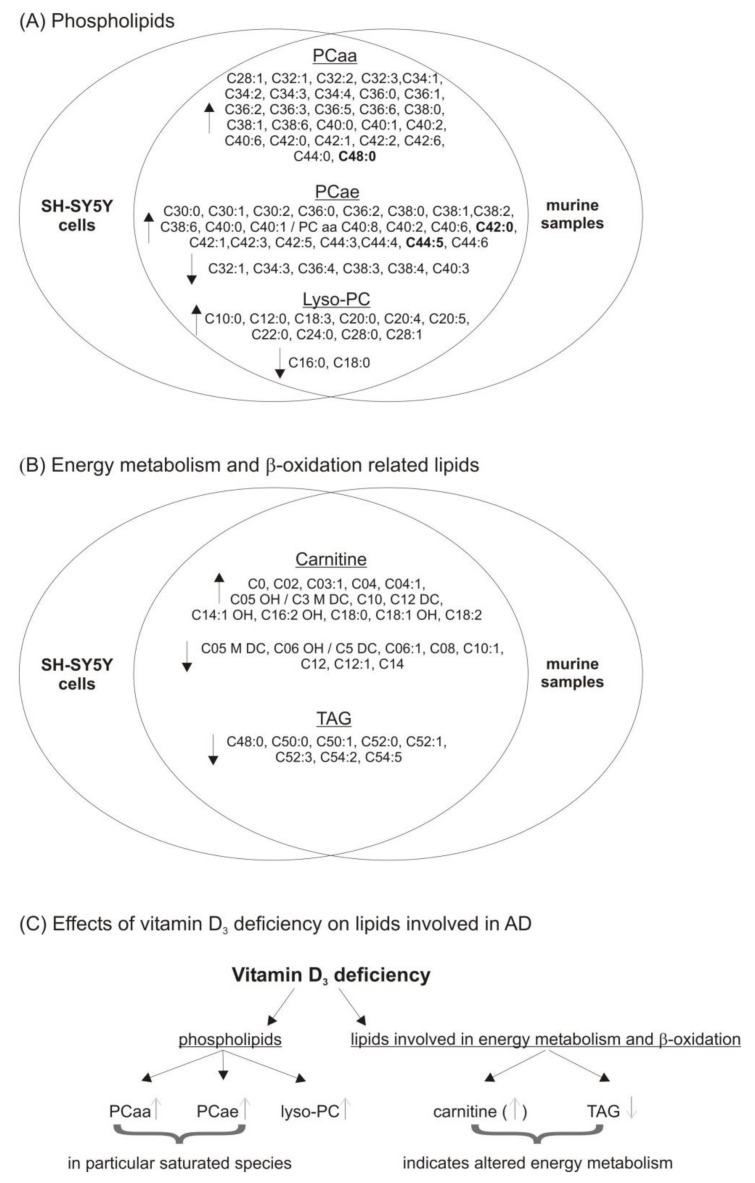
Overview of the effects of vitamin D_3_ hypovitaminosis on lipid metabolism in neuroblastoma cells and brain samples of mice with mild to moderate vitamin D deficiency. (**A**) Effects observed influencing the metabolism of phospholipids (phosphatidylcholine, PCaa; phosphatidylcholine plasmalogens, PCae; lyso-phosphatidylcholine, lyso-PC) in a Venn diagram. (**B**) The effect of vitamin D3 deficit on lipids involved in cellular energy metabolism and β-oxidation (carnitine and triacyl glycerides, TAG) in a Venn diagram. (**C**) Summary of the influence of vitamin D_3_ deficit on different lipid classes known to be affected in disorders such as neurodegenerative Alzheimer’s disease (AD).

In summary, both neuroblastoma cells and mouse brains showed vitamin D-dependent alterations in lipid homeostasis (see Figure 7). Most of the observed lipid changes occurred both in the cellular model of vitamin D_3_ deficiency and in vivo in the vitamin D_3_ deficient mouse model. Importantly, all observed changes in lipid species are discussed or known to have an impact on many neurological diseases. Several alterations in phospholipid homeostasis have been associated with neurodegeneration and therefore recommendations, in which lipids are beneficial with respect to neurodegenerative disease, have been reported in literature. To clarify whether our observed vitamin D_3_ deficient mediated lipid change might contribute to neurodegeneration, we summarized the phospholipids that were found in this study to be altered to the known changes in phospholipid homeostasis, which were associated with neurodegeneration (see Table 1).

Our results suggest that vitamin D is not only responsible for widespread regulation of related genes but also influences lipid homeostasis and in that way might also affect several diseases, in particular neurodegenerative diseases such as AD. Based on our findings, it can be summarized that the proposed mechanism from which lipid classes found to be influenced by vitamin D_3_ leading to AD pathology might be membrane fluidity, lipid raft formation, Aβ oligomer formation, oxidative stress and energy metabolism (see Table 1).

## Figures and Tables

**Table 1:** Summary of the phospholipids found in this study to be altered under hypovitaminosis D_3_ conditions to the known changes in phospholipid homeostasis associated with neurodegenerative Alzheimer’s disease.

Lipid Species	Vitamin D Deficiency	Selection of Proposed AD-Related Mechanisms
PUFA	↓	PUFAs are reported to be decreased in AD due to increased ROS levels. Moreover, in particular DHA is known to affect Aβ production via multiple pleiotropic mechanisms, including an increase of nonamyloidogenic processing by increase of ADAM17 protein levels accompanied by an increase in *ADAM17* expression, and a decrease in ADAM17 protein degradation. Furthermore, amyloidogenic APP processing is decreased in presence of PUFAs, resulting in reduced Aβ production. Underlying mechanisms include a decreased BACE1 activity; a changed distribution of BACE1 on the cell surface/intracellular BACE1 pool is discussed. With respect to γ-secretase, a direct effect of DHA on enzyme activity is reported, accompanied by a shift from γ-secretase from raft to nonraft [80]. Further potential mechanisms include effects on Aβ degradation. In particular, EPA has been shown to increase IDE mediated Aβ degradation [81]. Importantly, oxidized PUFA species seem to have controversial effects [82].
SFA	↑	Saturated fatty acids are reported to be linked with dementia in several studies, reviewed, e.g., in [83]. Potential molecular mechanisms might include an effect of SFA on α-secretase and membrane fluidity (SFAs show a decrease on α-secretase activity and membrane fluidity compared to MUFAs and PUFAs) [64]. In return, SFAs seem to increase β-amyloid secretion and are associated with reduced β-amyloid elimination. Moreover, SFAs are discussed to be associated with an increase in diabetes and insulin resistance, which play a crucial role in AD [84].
Lyso-PC	↑	Lyso-PCs are reported to be increased during aging and in particular in AD [43,85]. Importantly, a tight link between phospholipase A2, resulting in lyso-PC generation, and Aβ has been reported [86]. In line, PLA2 reduction was shown to ameliorate cognitive deficits in AD mouse models [87]. Additionally, lyso-PC increases neurotoxicity of Aβ_1-42_, and a potential impact of Aβ oligomerization induced by lyso PCs is discussed [74].
Plasmalogens	↑	Plasmalogens are known to be decreased in AD brains [88]. Plasmalogens are known to be vulnerable to ROS species, being increased in AD [89]. In return, plasmalogens decrease Aβ generation [90]. Importantly, vitamin D_3_ deficiency results in an increase in plasmalogens both in murine brain and in cell culture. Further experiments are needed to proof whether vitamin D_3_ supplementation in human results in decreased plasmalogens levels, which would be unfavorable with respect to AD. Therefore, an additional plasmalogens—in addition to vitamin D_3_ supplementation—might be useful and should be further investigated with respect to AD.

## Data Availability

Not applicable.

## Supplementary Materials

The following are available online at https://www.mdpi.com/article/10.3390/biom11111699/s1, Figure S1: Scheme of tested hypotheses relating to link between vitamin D_3_ and common pathologies. Figure S2: Cell viability, IL-34 expression, oxygen consumption and matrix effects.
