# Supplementary material for: Impact of Vitamin D3 Deficiency on Phosphatidylcholine-/Ethanolamine, Plasmalogen-, Lyso-Phosphatidylcholine-/Ethanolamine, Carnitine- and Triacyl Glyceride-Homeostasis in Neuroblastoma Cells and Murine Brain
Source: Biomolecules. 2021 Nov 15;11(11):1699. doi: 10.3390/biom11111699 (PMC8615687; doi:10.3390/biom11111699)
Supplement: Supplementary file 1 [file biomolecules-11-01699-s001.zip › biomolecules-1376909-supplementary.pdf]

Supplementary Materials

Impact of vitamin D3 deficiency on phosphatidylcholine- / ethanolamine, plasmalogen-, lyso-phosphatidylcholine- / ethanolamine, carnitine- and triacyl glyceride-homeostasis in neuroblastoma cells and murine brain

Anna Andrea Lauer [1], Lea Victoria Griebsch [1], Sabrina Melanie Pilz [1], Daniel Janitschke [1], Elena Leoni Theiss [1], Jörg Reichrath [2], Christian Herr [3], Christoph Beisswenger [3], Robert Bals [3], Teresa Giovanna Valencak [4,5], Dorothea Portius [6], Heike Sabine Grimm [1], Tobias Hartmann [7] and Marcus Otto Walter Grimm [1,7,8*]

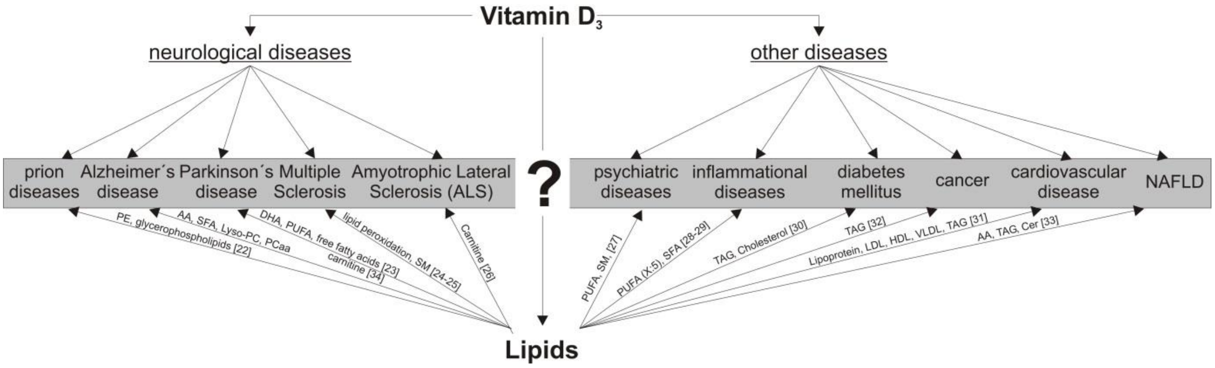

Figure S1. Scheme of tested hypotheses relating to link between vitamin D3 and common pathologies. An association of vitamin D3 with neurological and other diseases is well known in current literature. Moreover, the involvement of lipids in these disorders was shown. Based on this and the fact, that vitamin D3 is able to modulate the homeostasis of numerous lipids, the aim of this study was to investigate, if this secosteroid can trigger the pathophysiology of diseases, for example Alzheimer´s disease (AD), by affecting the involved lipids. PE: phosphatidylethanolamines; AA: arachidonic acid; SFA: saturated fatty acids; Lyso-PC: lyso-phosphatidylcholines; PCaa: phosphatidylcholines; DHA: docosahexaenoic acid; PUFA: poly-unsaturated fatty acids; SM: sphingomyelin; Cer: ceramide; TAG: triacyl glyceride; LDL: low density lipoprotein; HDL: high density lipoprotein; VLDL: very low density lipoprotein; NAFLD: non-alcoholic fatty liver disease.

Figure S2

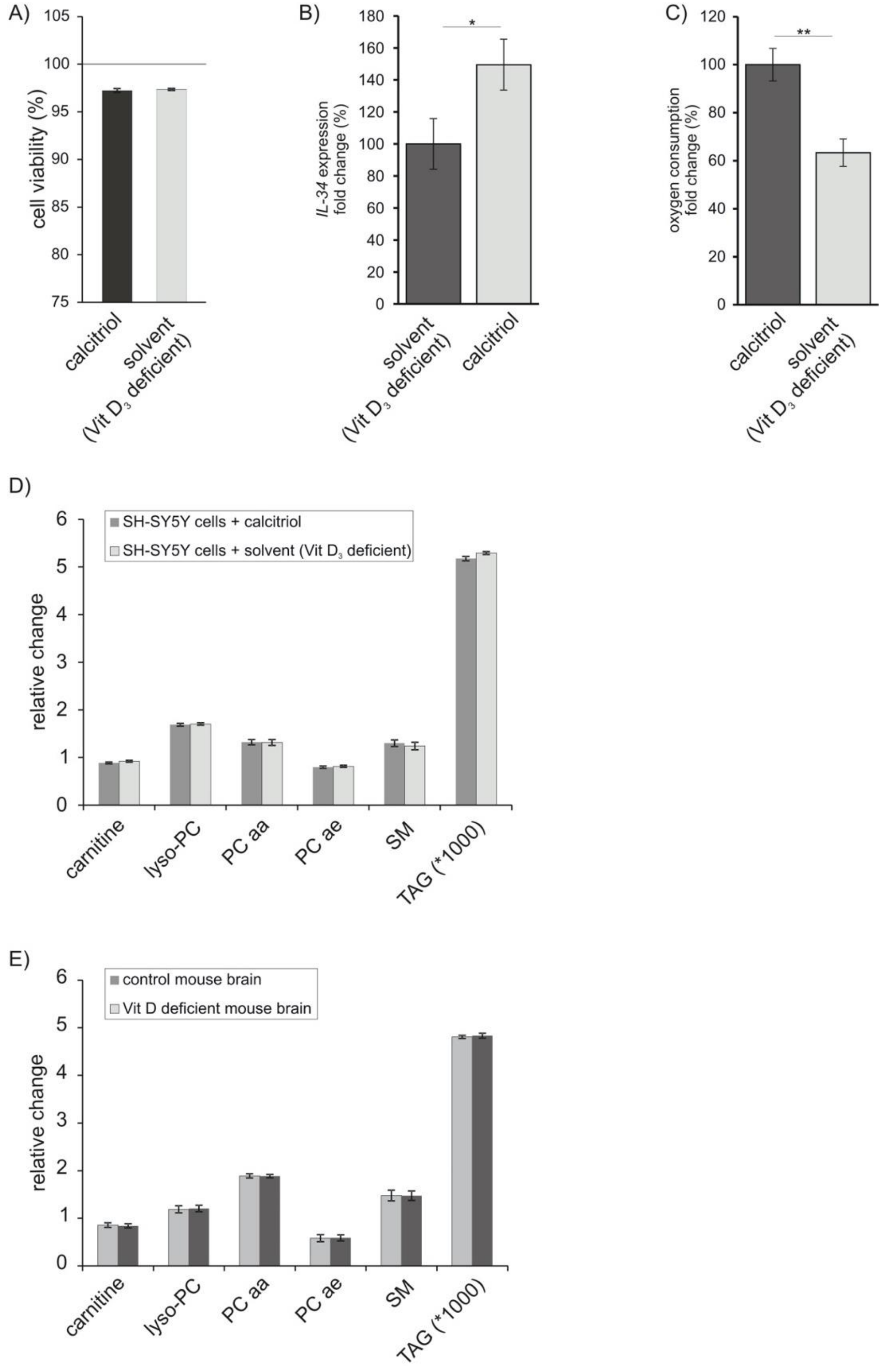

Figure S2. Cell viability, *IL-34* expression, oxygen consumption, and matrix effects. **(A)** Investigation of cell viability after incubation of SH-SY5Y cells with 100 nM 1,25-dihydroxyvitamin D_3 for 48 h via cytotoxicity detection assay. **(B)** Expression of *IL-34* in SH-SY5Y cells treated with 100 nM 1,25-dihydroxyvitamin D_3 for 48 h. Error bars represent the standard error of the mean (SEM) and significance was * $p \leq 0.05$. **(C)** Oxygen consumption in SH-SY5Y cells treated with 100 nM 1,25-dihydroxyvitamin D_3 for 48 h via Extracellular Oxygen Consumption Assay. Error bars represent the standard error of the mean (SEM) and significance was ** $p \leq 0.01$. **(D)** Calculated matrix effects of the analyzed lipid species carnitine, lyso-phosphatidylcholine (lyso-PC), phosphatidylcholine (PCaa), phosphatidylcholine-plasmalogen (PCae), sphingomyelin (SM) and triacyl glyceride (TAG) in calcitriol and solvent control treated SH-SY5Y cells. **(E)** Matrix effects in brain samples of vitamin D deficient and control mice. Error bars represent the standard error of the mean (SEM).
